# Applying ecological and evolutionary theory to cancer: a long and winding road

**DOI:** 10.1111/eva.12021

**Published:** 2012-11-16

**Authors:** Frédéric Thomas, Daniel Fisher, Philippe Fort, Jean-Pierre Marie, Simon Daoust, Benjamin Roche, Christoph Grunau, Céline Cosseau, Guillaume Mitta, Stephen Baghdiguian, François Rousset, Patrice Lassus, Eric Assenat, Damien Grégoire, Dorothée Missé, Alexander Lorz, Frédérique Billy, William Vainchenker, François Delhommeau, Serge Koscielny, Raphael Itzykson, Ruoping Tang, Fanny Fava, Annabelle Ballesta, Thomas Lepoutre, Liliana Krasinska, Vjekoslav Dulic, Peggy Raynaud, Philippe Blache, Corinne Quittau-Prevostel, Emmanuel Vignal, Hélène Trauchessec, Benoit Perthame, Jean Clairambault, Vitali Volpert, Eric Solary, Urszula Hibner, Michael E Hochberg

**Affiliations:** 1 MIVEGEC (UMR CNRS/IRD/UM1) 5290Montpellier Cedex 5, France; 2 CREECMontpellier Cedex 5, France; 3 IGMM, CNRS UMR 5535Montpellier, France; 4 Université Montpellier 1Montpellier, France; 5 Université Montpellier 2Montpellier, France; 6 CRBM, CNRS UMR 5237Montpellier, France; 7 UPMC, UMRs872 and Saint-Antoine HospitalAP-HP Paris, France; 8 UMMISCO (UMI IRD/UPMC)Bondy Cedex, France; 9 UPVDPerpignan, France; 10 CNRS, UMR 5244, Ecologie et Evolution des Interactions (2EI)Perpignan, France; 11 ISEM, CNRSMontpellier Cedex, France; 12 INRIA Paris-Rocquencourt, CNRS UMR 7598 and UPMC Laboratoire Jacques-Louis LionsParis cedex 05, France; 13 INSERM UMR 1009, Institut Gustave RoussyVillejuif, France; 14 UPMC Univ Paris 06Paris, France; 15 AP-HP, Hôpital Saint-AntoineParis, France; 16 CNRS – INRIA UMR 5208 Institut Camille Jordan, Université Lyon 1Lyon, France; 17 The Santa Fe InstituteSanta Fe, NM, USA

**Keywords:** cancer, disease biology, evolutionary medicine, evolutionary theory

## Abstract

Since the mid 1970s, cancer has been described as a process of Darwinian evolution, with somatic cellular selection and evolution being the fundamental processes leading to malignancy and its many manifestations (neoangiogenesis, evasion of the immune system, metastasis, and resistance to therapies). Historically, little attention has been placed on applications of evolutionary biology to understanding and controlling neoplastic progression and to prevent therapeutic failures. This is now beginning to change, and there is a growing international interest in the interface between cancer and evolutionary biology. The objective of this introduction is first to describe the basic ideas and concepts linking evolutionary biology to cancer. We then present four major fronts where the evolutionary perspective is most developed, namely *laboratory and clinical models*, *mathematical models*, *databases*, and *techniques and assays*. Finally, we discuss several of the most promising challenges and future prospects in this interdisciplinary research direction in the war against cancer.

## Introduction

In 1971, the US president Richard Nixon declared the now famous ‘war on cancer’, predicting victory within 5 years. Forty years later, cancer still accounts for about one-quarter of human deaths in wealthy countries and about one-eighth worldwide (World Health Organization 2008). Despite significant progress, treatments have not met expectations and cancer research is now at a crossroad, needing new ideas, major innovation, and new and unprecedented transdisciplinary teams of scientists (Drake 2011). Although the theory of cancer initiation and progression is deeply rooted in evolutionary and ecological concepts (Cairns 1975; Nowell 1976), many promising opportunities for the application of evolutionary biology to oncology remain unexplored. To what extent does evolutionary theory provide a useful framework for understanding and predicting cancer progression in laboratory and clinical settings, and is it more applicable to certain cancers rather than others? What level of mathematical sophistication is necessary to investigate observations and will this require stochastic components, meaning less predictability? Can we alter the competition between cancerous and healthy cells by boosting the fitness of benign cells? What are the selective effects of therapies?

The important challenges in cancer research are to understand susceptibility, emergence, and progression, and to predict treatment outcomes, including the major problem of relapse, given both limited individual-level information and data from past clinical cases and laboratory studies. Whereas enormous progress has been made in understanding cell-autonomous molecular mechanisms of oncogenic transformation, our vision of tumor–host interactions is still in its infancy. Specifically, we know that the vast majority of cells that have initiated an oncogenic transformation will be eliminated by the host, but we lack the capacity to predict which will escape the surveillance mechanisms (Folkman and Kalluri 2004; Bissell and Hines 2011). Our increased capacity to detect precancerous lesions and circulating or dormant tumor cells is, thus, difficult to put into practice. Indeed, as exemplified by a recent controversy regarding early diagnosis of prostate cancer (Cooperberg et al. 2011; see also Epstein et al. 2001 for breast cancer), a strategy to aggressively target all precancerous or dormant cancerous cells carries the risk of overtreatment, when active surveillance may be a safer and acceptable alternative. Thus, while we need further advances in the description, classification, and understanding of molecular mechanisms of cancer, we must strive to meet additional challenges, such as, for instance, our capacity to predict and model the interrelationships between the tumor and its environment at different scales (Bissell and Hines 2011).

While defining the probability of progression of early lesions to full-blown cancer is a major challenge in cancer research, it must be emphasized that today most patients are diagnosed when their disease is at an advanced, metastatic stage (reviewed in Valastyan and Weinberg 2011). From the clinical standpoint, it is thus of the utmost importance to develop efficient therapies to fight tumor cell proliferation with minimal side effects and to either manage or prevent the emergence of resistance in neoplastic cell populations. In this respect, ecological and evolutionary approaches have contributed with mathematical models (Gatenby and Vincent 2003; Komarova and Wodarz 2005; Foo and Michor 2009; Gatenby et al. 2009; Cunningham et al. 2011; Lorz et al. in press; Hochberg et al. this volume) yielding predictions such as (i) evolving neoplasms and microenvironments may thwart cell-targeted therapies (Gillies et al. 2012), (ii) multiple therapeutic targets are less likely to result in resistance than monotherapies (Komarova and Wodarz 2005; Lorz et al. in press), and (iii) developing preventive therapies with minimal side effects may control or eliminate incipient lesions and neoplasms, and prevent the emergence of chemoresistant lineages (Hochberg et al. this volume).

The objective of this special issue is to continue the construction of a broad base for a more balanced approach to cancer research, by assembling some of the latest, most exciting results, syntheses, and perspectives relating to the action of natural selection and drift in determining evolutionary dynamics and emergent patterns in cancer. Emergent patterns include, but are not limited to, interspecific differences in cancer susceptibility and tumor suppression, cancer initiation, and progression, and cancer therapies.

## Evolutionary biology in the study of cancer

An important conceptual breakthrough in understanding cancer lies in Darwinian and ecological theories: cancer is a disease of opportunity, associated with clonal evolution, expansion, and competition within the body (Cahill et al. 1999; Merlo et al. 2006; Greaves and Maley 2012). Specifically, somatic cellular selection and evolution are the fundamental processes leading to malignancy, metastasis, and resistance to therapies, with the contribution of cancer stem cells as the progenitors of these more differentiated cell types (Shipitsin and Polyak 2008; Greaves this volume). However, it is not known whether patients relapse because cancer stem cells are intrinsically resistant to therapy, and/or because therapy selects for resistance or, most likely, both. An additional complication is the tremendous plasticity of cancer cells and their ability to acquire stem cell characteristics through deregulated expression of just a few genes (Takahashi and Yamanaka 2006; Mani et al. 2008; Morel et al. 2008). This phenomenon notwithstanding, tumors can be viewed as collections of individuals (cells) that accumulate genetic and epigenetic changes, and through their interactions with the environment (selection), adaptively evolve. Examples include stressful microenvironments affecting the evolution of the invasive phenotypes (Lee et al. 2011), and the evolution of resistance to toxicity during tumor growth, providing a competitive advantage with respect to wild-type cells (Gatenby et al. 2006; Vineis and Berwick 2006). Epigenetic changes have been recognized as neoplasm markers since the 1980s (Romanov and Vanyushin 1981); however, their role is still elusive. Some see them as a byproduct of deregulated gene expression, others as initial event in oncogenesis. Only very recently, epimutations were included in mathematical models that attempt to describe the evolution of tumors (Iwami et al. 2012). Transient increases in epigenetic mutations as a result of stressful environments could provide a solution to the conundrum that rapid evolution of somatic cells into neoplasms would require very high mutation rates, placing them at risk of extinction due to excessive levels of (epi)genetic instability (Cahill et al. 1999; Solé and Deisboeck 2004).

Based largely on cytological, molecular, and genetic studies, researchers have recently argued that cancers should be viewed both as genetically and phenotypically heterogeneous populations *within* individuals (Marusyk and Polyak 2010) and as different ‘species’ *between* individuals (Merlo and Maley 2010; Gatenby 2011). This variability suggests that stochastic and complex interactive forces reduce our ability to make generalizations about different stages in carcinogenesis. Mechanisms that could generate this variability are mutations, chromosomal damage, including catastrophic events such as recently described chromotripsis (Stephens et al. 2011), deletions and duplications, heritable changes in gene expression, DNA methylation, and changes in protein conformation (Maley et al. 2006). Interestingly, recent sequence data from a large number of different tumor types have revealed the frequent occurrence of events affecting global control of cellular functions, for instance, changes in chromatin modifications or RNA processing (recently reviewed in the study by Ma et al. 2012), likely to impact the rate of tumor evolution. Tarafa et al. (2003) showed that chromosomal instability arises early in cancer progression, and that major genetic changes tend to occur in one of a few particular orders, meaning that some level of predictability in key events may be possible for some cancers. Progress in phylogenetic reconstruction (Gerlinger et al. 2012), inference methods (Riester et al. 2010), agent based modelling (Sprouffske et al. 2011), and whole genome sequencing (Parmigiani et al. 2009) will be key to untangling and reconstructing somatic evolutionary pathways. A major unresolved question, therefore, is whether a single overarching framework can incorporate observed variability and make cancer a more predictable phenomenon both at individual and population levels.

Evolutionary and ecological theory has already proven useful in our understanding of cancer, but many basic parts of the puzzle are missing. We need to know how variation is created and selected for, and the adaptive consequences of interactions between environments and genes. We also need to understand the relative roles of stem cells and differentiated cells in cancer dynamics (Visvader and Lindeman 2008; Greaves this volume), taking into account the cellular plasticity that might render the distinction between the two largely artificial. Perhaps, the greatest challenge is to understand the relevance of processes occurring at one scale for patterns at another (Tomlinson and Bodmer 1999). For example, it is increasingly recognized that many cancers are associated with chronic inflammation, aging, and changes in local microenvironments, and in tissue structure and architecture (Polyak et al. 2009; Bissell and Hines 2011; Gatenby 2011). Are these phenomena causes and/or consequences of genetic instability and progression, or less interestingly, correlations without demonstrable causation?

Evaluating current theories and advancing new hypotheses will require use of the latest techniques and the development of new approaches. Four major fronts have proved indispensable to cancer research.

### Laboratory and clinical models

There is ample evidence of intratumor clonal heterogeneity in human cancer (recently reviewed in Marusyk et al. 2012), with many examples from hematopoietic malignancies and from solid tumors. There are ongoing efforts worldwide to provide a comprehensive description of genomic, transcriptomic, and epigenomic profiles of a large number of tumors (International Cancer Genome Consortium 2010). Whereas such global analyses will undoubtedly lead to a better understanding of the complexity of tumor cell populations, there is also a need for model systems that are simpler to analyze and possible to manipulate. The simplest of such models consists of analyzing two or more genetically distinct tumor clones grown in coculture under different environmental constraints. More ambitiously, the tumor microenvironment can be mimicked in culture, for example, by constructing three-dimensional models with controlled physicochemical characteristics of the extracellular matrix (reviewed in Egeblad et al. 2010). Finally, *in vivo* animal models are particularly attractive for studies of tumor growth and evolution. For example, mammary tumors inoculated into cleared fat pads (Mani et al. 2008; Fridriksdottir et al. 2011) or hepatic tumorigenesis following intrasplenic injection of transformed cells (Zender et al. 2006) have already provided a wealth of information. Such models carry rich opportunities for studying complex interactions between cell clones.

While cellular competition is the issue most often addressed, other types of interactions, with possible major clinical impacts have also been observed. For example, it has recently been reported that an apoptosis proficient clone provides an advantage to a tumor relapse through stimulation of growth via paracrine signaling from cells dying after radiation therapy (Huang et al. 2011). From an evolutionary perspective, however, an additional complication comes from a recent finding that subtle differences in the precise mechanism of programmed cell death give rise to diametrically opposed consequences of the host immune reaction toward the dying cells (Green et al. 2009). Understanding how selection drives the evolution of complex interactions between cell clones remains a challenging question.

### Mathematical models

Mathematical models are important tools for characterizing the complexity of cancerogenesis and the underlying role of evolutionary processes (Foo and Leder this volume). There are various ways for models to include the rich and puzzling biological diversity that may be observed in tumor spatial and temporal heterogeneity, and whenever possible to identify evolutionary trends, aiming at prediction and control, in particular, for practical therapeutic purposes in oncology.

Nevertheless, computer simulations are never proofs, even though they can give hints to what can actually be proven by mathematics. The simplest mathematical models aim at describing a well-characterized situation by sets of ordinary differential equations or probabilistic branching processes (Iwasa et al. 2006; Foo and Michor 2009; Tomasetti and Levy 2010) and yield answers to important problems such as the prediction of drug resistance. As regards more physiologically detailed models designed to predict cell population behavior, there are two main streams of models: agent-based stochastic models and spatially or physiologically structured continuous deterministic models. Agent-based models (Anderson et al. 2006) include stochastic rules that decide how a whole cell population passes from stage *i* to stage *i* + 1, by taking into account all individual cell behaviors. Spatially or physiologically structured continuous deterministic population models are based on partial differential or integrodifferential equations and consider the population as a whole, structured according to a continuous variable that can be space (Frieboes et al. 2006, 2009), but also molecular content in a protein, or level of expression of a phenotypic trait (Lorz et al. 2012), etc., that has been selected as relevant to describe population heterogeneity. Continuous deterministic population models are not limited by computer performance, which is not the case for agent-based models; conversely, agent-based models are more flexible than simpler deterministic models and may take into account virtually any local biological phenomenon. Comparisons between the two approaches have been made (Byrne and Drasdo 2009; Osborne et al. 2010), pointing out their respective advantages and drawbacks.

Models of these different types have simulated a range of the many facets of tumor biology, for example, stem cell dynamics (van Leeuwen et al. 2007; Michor 2008; Sottoriva et al. 2011), the stochastic emergence of resistance during clonal expansion (Iwasa et al. 2006), the numerical dynamics of differentiated cell types (Dingli et al. 2007), metastasis (Gatenby and Vincent 2003; Frieboes et al. 2006; Michor et al. 2006), and therapeutic outcomes (Michor et al. 2005; Frieboes et al. 2009; Gatenby et al. 2009). These models have been successful in evaluating hypothetical scenarios in adaptation to different microenvironmental challenges, such as hypoxia and acidosis (Anderson et al. 2006; Gatenby et al. 2006), and to chemotherapy (Michor et al. 2005; Foo and Michor 2009; Cunningham et al. 2011). There has been a recent surge in models integrating observations and experimental data (Frieboes et al. 2009; Bozic et al. 2010; Byrne 2010) and next-generation models capable of simulating highly detailed somatic genetic events (Stephan-Otto Attolini et al. 2010; Sprouffske et al. 2011).

### Databases

Numerous international and national databases are now available online relating to several socioeconomic and health topics, including statistics on cancer incidence and mortality worldwide, or cancer mutations and copy number variation (CNV; International Agency for Research on Cancer (IARC GLOBOCAN project, 2008, http://globocan.iarc.fr/; http://www.biologie.uni-hamburg.de/b-online/library/genomeweb/GenomeWeb/human-gen-db-mutation.html; http://gwas.biosciencedbc.jp/cgi-bin/cnvdb/cnv_top.cgi). Such databases provide a unique opportunity to conduct comparative analyses at the largest scale to explore or validate various hypotheses on cancer origin and/or dynamics. For instance, macroecological approaches have been recently employed to study the infectious causation of certain cancers (Thomas et al. 2011, 2012a; Vittecoq et al. 2012) or to explore the evolutionary links between malignancies and birth weight (Thomas et al. 2012b).

### Techniques and assays

Techniques originally used to reconstruct the evolutionary history of species have been applied to tracing the somatic lineages of healthy and cancerous cells within an individual. State of the art genetic, molecular, cellular, and biochemical technologies have been applied to study tumor clonality, plasticity and, evolution. This is in fact a major issue for tumor characterization, due to problems of representative sampling (Marusyk and Polyak 2010; Navin et al. 2011). Currently, major progress in tumor characterization comes from the generalized use of global genomic and postgenomic analyses (next-generation sequencing, transcriptomics, proteomics). Two complementary strategies are currently being developed to address tumor heterogeneity: multiple comparisons of single cells from macrodissected tumors (Navin et al. 2010, 2011) and the development of analytical methods to infer cell populations from unmixed tumor-wide gene expression or SNP data (Subramanian et al. 2012). While these technologies have become widely available and technically reliable, their usefulness is largely determined by careful definition of biological samples and models under study. Apart from cancer cell lines, this research relies on human fresh tumor samples being frozen and stored in highly specialized biorepositories. To be useful, samples need to be linked with clinical data, and this is a major challenge given the complexity of different ethical laws in different countries (Riegman and van Veen 2011).

## Challenges and future prospects

Ecology and evolutionary biology as scientific fields have, until now, developed in relative isolation from the health sciences. This is unfortunate because links between these areas have the potential to reveal new perspectives and avenues for fundamental research (Daoust et al., Khalid et al. this volume). Evolutionary processes and their relevance to the biology and epidemiology of disease also hold the promise of instructing on more applied health issues, offering scientific reasons for why certain medical approaches are more successful than others. Understanding basic scientific processes could be translated into huge progress for therapies. For instance, understanding the proliferative and survival effects due to chimeric tyrosine kinase (bcr-abl) in Chronic Myelogenous Leukemia has led to a targeted therapy, transforming the prognosis of this lethal disease. Similarly, systematic screening for acquisition of further bcr-abl mutations permitted to anticipate escape of subclones and rapidly administer second generation TK inhibitors, thus restoring drug sensitivity (Gibbons et al. 2012). The traditional separation between subdisciplines is a fundamental limitation that needs to be overcome if complex processes, like oncogenesis, are to be understood. Below, we present some of the most important current challenges amenable to ecological and evolutionary approaches to understand major fundamental and therapeutic aspects of cancer. Recent reviews on these and related topics can be found in the studies by Merlo et al. (2006), Pienta et al. (2008), Gerlinger and Swanton (2010), Caulin and Maley (2011), Greaves and Maley (2012), Aktipis et al. (2011).

### Ecology: infectious agents and cancer

The World Health Organization currently estimates that 20% of cancers are caused by infectious agents, with special emphasis on viruses and bacteria. Identifying infectious agents that directly or indirectly contribute to oncogenesis remains a priority in the war on cancer for an obvious reason: insofar as infectious diseases are preventable or treatable, cancers associated with infection could be preventable as well (De Martel and Franceschi 2009; Ewald and Ewald this volume). Persistent infections may promote cancer because long-term host defensive responses induce inflammation that subsequently increases mutation rates (Fitzpatrick 2001). In addition, intracellular pathogens may manipulate their host cells in ways that disrupt traditional cell barriers to cancer, allowing oncogenic mutations to accumulate through time. Current evidence links Epstein–Barr virus, Hepatitis B and C viruses, the bacteria *Helicobacter pylori*, human papilloma virus, and the trematodes *Schistosoma haematobium, S. japonicum*, *and S. mansoni* to cancers of the lymph nodes, liver, stomach, cervix, bladder, colon, and liver, respectively. The complete list of oncogenic pathogens is probably far from being fully established (Ewald 2009; zur Hausen 2009; Dapito et al. 2012), and certain scientists speculate that most cancers may have an infectious origin (Ewald 2009).

### Evolution: selection for cancer suppression at the organism level

Why are some species and/or individuals more at risk than others to particular cancers? Answering this question necessitates we understand the ecological and evolutionary bases of cancer vulnerabilities. Comparing cancer incidence among wildlife species is currently considered a promising research direction to highlight the natural defenses against cancers retained by natural selection, and to ultimately improve cancer prevention in humans (Caulin and Maley 2011). This requires that we resolve Peto's paradox (Peto et al. 1975): the lack of a correlation between body size (or longevity) and cancer across species (Roche et al. this volume; Nunney, this volume). If each dividing cell in a multicellular organism has the same probability of initiating a malignant neoplasm, then all else being equal, the more cells an organism has, the greater the chance of a cancer emerging. Moreover, transitions to malignancy are expected to increase with the number of cell divisions – that is with organism lifespan. Numerous studies have shown correlations between longevity and body size, making Peto's paradox all the more difficult to resolve (Caulin and Maley 2011). How can large, long-lived organisms avoid the emergence of cancer or overcome its progression should it emerge? Few empirical studies have addressed this question (Seluanov et al. 2008; Gorbunova and Seluanov 2009). There is also a crucial need to develop mathematical models to explore theoretically how cancer vulnerability among wildlife species could have been shaped by natural selection. The question is by far more complex than just a problem of body size and longevity – instead it undoubtedly also depends on the relative importance to fitness of cancer, infectious and parasitic diseases, predation, and adverse environmental conditions (Roche et al. in press). Considering these wildlife species within their ecosystem could be the missing ingredient to resolve Peto's paradox and give critical insights into mechanisms of cancer resistance. Besides the possible application for improving cancer prevention for humans, modeling approaches are a promising way forward for understanding the impact of cancer in wildlife conservation.

Recent advances have also highlighted that cancer-causing genes can be maintained in populations through various processes. For instance, as for genes reducing survival in general, natural selection is unlikely to strongly counter-select oncogenes when their negative effects occur after reproduction (Frank 2004, 2005; Balducci and Ershler 2005). Additionally, antagonistic pleiotropy might be an important component in the evolutionary maintenance of oncogenes (for a review, see Crespi and Summers 2006). Certain genes indeed have beneficial effects in early life, when natural selection is strong, but harmful at later ages, when the effect of selection on evolutionary adaptation weakens. In the context of cancer, this phenomenon - antagonistic pleiotropy - has been found in animal models, perhaps the most stunning case being *Xiphophorus* fish, where late life melanoma-promoting oncogene alleles are associated with early life advantages (Fernandez and Bowser 2010). There is increasing evidence that some human cancers occurring at later ages may result from negative trade-offs with early age adaptations (Summers and Crespi 2008, 2010; Smith et al. 2012), such as, for instance, high birth weight – a life-history trait that has a genetic basis and is also associated with fitness benefits early in life (e.g., survival until maturity, Thomas et al. 2012b; Smith et al. 2012).

### Evolution: within-organism selection for cancer cells

Given mounting evidence that neoplasms are characterized by increased mutation rates, chromosomal anomalies, and epigenetic alterations, applying evolutionary thinking to study the proliferation of cancerous cells is crucial to understanding neoplastic progression. The fitness of neoplastic cells is shaped by various factors, ranging from the quantity and the quality of genetic and epigenetic alterations that are beneficial to a neoplastic clone and also by the interactions with cells and other factors from the local environment. One of the most promising models for understanding the role of natural selection in cancer progression is myeloid malignancies. There may be less genetic variation and more likelihood for successful treatments leading to eradication, or at least cancer management. Of the many mutations that characterize myeloid malignancies, some (*TET2*, *ASXL1*) can initiate a preleukemic clone, whereas others (*MPL, JAK2*) are phenotypic lesions that trigger the overt malignant disease (Vainchenker et al. 2011). A preleukemic state may also be the consequence of germ-line mutations. Thus two preleukemic contexts, either clonal or polyclonal, driven by somatic or germ-line lesions, may exist. The degree of overlap between them is difficult to assess, and initiating events may have subtle phenotypic consequences for years or decades, before the actual onset of a full-blown malignancy.

Evolution in solid tumors can differ substantially from that observed in myeloid cancers. Recent studies have analyzed the genomic landscape of human colorectal cancers and identified ∼80 nonsilent mutations in individual tumors, among which <15 were likely to be responsible for driving the initiation, progression, or maintenance of the tumor (Sjöblom et al. 2006; Wood et al. 2007; Leary et al. 2008; Cancer Genome Atlas Network 2012). These seminal studies concluded that although alterations in Wnt, K-Ras, and p53 pathways remain pivotal to tumor formation, a large number of mutations – each associated with a small fitness advantage – are likely to be involved in tumor progression. Future studies will need to consider several additional, important aspects of evolution in carcinogenesis, such as (i) When do selected ‘driver’ mutations (Bozic et al. 2010, Reiter et al. this volume) occur during tumor formation? (ii) What are the associated qualitative and quantitative changes in gene expression? (iii) Which genes/pathways are selected or counter-selected for during tumor development?

A major challenge in understanding cancerogenesis is relating process to pattern in malignant and preneoplastic lesions to untangle the dynamics of cell–cell competition. This will involve predicting how the host and the growing neoplasm, which change in time and spatially, affect signaling networks and have emergent impacts on the demography and evolution of progressing cancerous growth. This phenomenon is driven by expression of certain oncogenes, such as Myc, or tumor suppressors. This results in overgrowth or in active killing (induction of apoptosis) of ‘loser’ clone cells by the ‘winner’ clone and thus evolution. While the role of ‘active competition’ in tumorigenesis has yet to be demonstrated formally, it has been confirmed both in the context of mammalian tissue regeneration and in coculture of mammary carcinoma cell lines (Oertel et al. 2006; Tamori et al. 2010). Specifically, early work on breast epithelial cancer cell lines derived from a single spontaneous tumor arising in a Balb/C mouse demonstrated that the ‘winner’ phenotype of two related cell lines is independent of their intrinsic proliferative capacity when cultured alone (Miller et al. 1988). These results were obtained before our current understanding of apoptosis and of the role of active cellular competition. They merit reinvestigation in the context of evolutionary processes.

### Evolution: selection for therapeutic resistance during treatment

Clonal evolution not only selects for increased proliferation and survival, but is also instrumental in leading to invasion, metastasis, and therapeutic resistance. Understanding the costs and benefits of cellular resistance to therapeutic environments will constitute a major step forward in improving treatment outcomes (Martinez-Quintanilla et al. 2009). A hallmark of myeloid cancers is the ability of the malignant clone to evolve into multiple, frequently more aggressive subclones, as a result of either the natural history of the disease or the selective pressure of chemotherapy. In acute myeloid leukemia AML, several pathways involving the control of proliferation, apoptosis, and chemoresistance can participate in clonal evolution. Modifications in proliferation advantage have been related to ID1, a common target of activated tyrosine kinases in chronic and acute myeloid malignancies (Tang et al. 2009) while leukemic stem cells express high levels of various ABC proteins (ABCB1, ABCG2, ABCG1) that protect them from xenobiotics (Marzac et al. 2011). Other promising directions exist. For instance, cyclin-dependent kinases (CDK) are a new class of therapeutic targets for cancer cells, because they are required for cell proliferation and are efficiently inhibited by specific pharmacological agents (Hanahan and Weinberg 2011). Most importantly, unlike proliferation arrest induced by DNA damage (irradiation, genotoxic agents), which may stimulate cancer progression, the cytostatic effect of CDK inhibitors is direct and, a priori, free of such undesired side effects. However, as with all chemotherapeutic agents, the development of clinical resistance to CDK inhibitors is likely, and it would be necessary to investigate the evolution of this resistance. This is sketched in a theoretical way in the study by Lorz et al. (2012), where it is shown (by using an adaptive dynamic cell population model designed to study the evolution of drug resistance) that it is theoretically possible to overcome resistance in cancer cell populations (and to eradicate them with minimal damage to healthy cell populations) by using a combination of cytotoxic and cytostatic drugs.

## Concluding remarks

Ecology and evolution provide a framework for predicting cancer emergence, progression, and therapies. Phenotypic evolution of cancers will depend on the complexities of gene expression (e.g., pleiotropy and epistasis), epigenetic alterations, and cellular plasticity, all of which interact with the microenvironment. We can advance toward a predictive science for cancer if we can characterize and measure (i) demographic parameters (birth and death rates), (ii) potential (epi)genetic states, their relative fitnesses, and costs in different microenvironments, and their sequence of probable appearance, (iii) cellular and tissue functions (e.g., potential for motility and therefore metastasis), and (iv) genetic instabilities (aneuploidy, mutation rates). Historically, little attention has been focused on applications of evolutionary biology to understand and control neoplastic progression and to prevent therapeutic failures (Aktipis et al. 2011). We believe that an accurate evolutionary approach should unite and explain, rather than replace, the insights into mechanistic nonevolutionary studies. With this goal in mind, we are convinced that the topic ‘evolution and cancer’ is one of the most exciting and challenging research directions in the effort to understand multicellular organization and regulation, as well as in applying insights gained in the ‘war against cancer’.

## References

[b1] Aktipis CA, Kwan VS, Johnson KA, Neuberg SL, Maley CC (2011). Overlooking evolution: a systematic analysis of cancer relapse and therapeutic resistance research. PLoS ONE.

[b2] Anderson AR, Weaver AM, Quaranta V (2006). Tumor morphology and phenotypic evolution driven by selective pressure from the microenvironment. Cell.

[b3] Balducci L, Ershler WB (2005). Cancer and ageing: a nexus at several levels. Nature Review Cancer.

[b4] Bissell MJ, Hines WC (2011). Why don't we get more cancer? A proposed role of the microenvironment in restraining cancer progression. Nature Medicine.

[b5] Bozic I, Antal T, Ohtsuki H, Carter H, Kim D, Chen S, Karchin R (2010). Accumulation of driver and passenger mutations during tumor progression. Proceedings of the National Academy of Sciences of the United States of America.

[b6] Byrne HM (2010). Using mathematics to dissect cancer. Nature Reviews Cancer.

[b7] Byrne HM, Drasdo D (2009). Individual-based and continuum models of growing cell populations: a comparison. Journal of Mathematical Biology.

[b8] Cahill DP, Kinzler KW, Vogelstein B, Lengauer C (1999). Genetic instability and Darwinian selection in tumours. Trends in Cell Biology.

[b9] Cairns J (1975). Mutation selection and the natural history of cancer. Nature.

[b10] Cancer Genome Atlas Network (2012). Comprehensive molecular characterization of human colon and rectal cancer. Nature.

[b11] Caulin AF, Maley CC (2011). Peto's paradox: evolution's prescription for cancer prevention. Trends in Ecology and Evolution.

[b12] Cooperberg MR, Carroll PR, Klotz L (2011). Active surveillance for prostate cancer: progress and promise. Journal of Clinical Oncology.

[b13] Crespi B, Summers K (2006). Positive selection in the evolution of cancer. Biological Review.

[b14] Cunningham JJ, Gatenby RA, Brown JS (2011). Evolutionary dynamics in cancer therapy. Molecular Pharmacology.

[b15] Dapito DH, Mencin A, Gwak GY, Pradere JP, Jang MK, Mederacke I, Caviglia JM (2012). Promotion of hepatocellular carcinoma by the intestinal Microbiota and TLR4. Cancer Cell.

[b16] De Martel C, Franceschi S (2009). Infections and cancer: established associations and new hypotheses. Critical Reviews in Oncology and Haematology.

[b17] Dingli D, Traulsen A, Michor F (2007). Symmetric stem cell replication and cancer. PLoS Computational Biology.

[b18] Drake N (2011). Forty years on from Nixon's war, cancer research ‘evolves’. Nature Medicine.

[b19] Egeblad M, Rasch MG, Weaver VM (2010). Dynamic interplay between the collagen scaffold and tumor evolution. Current Opinion in Cell Biology.

[b20] Epstein SS, Bertell R, Seaman B (2001). Dangers and unreliability of mammography: breast examination is a safe, effective, and practical alternative. International Journal of Health Services.

[b21] Ewald PW (2009). An evolutionary perspective on parasitism as a cause of cancer?. Advances in Parasitology.

[b22] Fernandez AA, Bowser PR (2010). Selection for a dominant oncogene and lage male size as a risk factor for melanoma in the Xiphophorus animal model. Molecular Ecology.

[b23] Fitzpatrick FA (2001). Inflammation, carcinogenesis and cancer. International Immunopharmacology.

[b24] Folkman J, Kalluri R (2004). Cancer without disease. Nature.

[b25] Foo J, Michor F (2009). Evolution of resistance to targeted anti-cancer therapies during continuous and pulsed administration strategies. PLoS Computational Biology.

[b26] Frank SA (2004). Age-specific acceleration of cancer. Current Biology.

[b27] Frank SA (2005). Age-specific incidence of inherited versus sporadic cancers: a test of the multistage theory of carcinogenesis. Proceedings of the National Academy of Sciences of the United States of America.

[b28] Fridriksdottir AJR, Petersen OW, Rønnov-Jessen L (2011). Mammary gland stem cells: current status and future challenges. The International Journal of Developmental Biology.

[b29] Frieboes HB, Zheng X, Sun C-H, Tromberg B, Gatenby R, Cristini V (2006). An integrated computational/experimental model of tumor invasion. Cancer Research.

[b30] Frieboes HB, Edgerton ME, Fruehauf JP, Rose FR, Worrall LK, Gatenby RA, Ferrari M (2009). Prediction of drug response in breast cancer using integrative experimental/computational modeling. Cancer Research.

[b31] Gatenby RA (2011). Of cancer and cave fish. Nature Review Cancer.

[b32] Gatenby RA, Vincent TL (2003). An evolutionary model of carcinogenesis. Cancer Research.

[b33] Gatenby RA, Gawlinski ET, Gmitro AF, Kaylor B, Gillies RJ (2006). Acid-mediated tumor invasion: a multidisciplinary study. Cancer Research.

[b34] Gatenby RA, Silva AS, Gillies RJ, Frieden BR (2009). Adaptive therapy. Cancer Research.

[b35] Gerlinger M, Swanton C (2010). How Darwinian models inform therapeutic failure initiated by clonal heterogeneity in cancer medicine. British Journal of Cancer.

[b36] Gerlinger M, Rowan AJ, Horswell S, Larkin J, Endesfelder D, Gronroos E, Martinez P (2012). Intratumor heterogeneity and branched evolution revealed by multiregion sequencing. The New England Journal of Medicine.

[b37] Gibbons DL, Pricl S, Kantarjian H, Cortes J, Quintás-Cardama A (2012). The rise and fall of gatekeeper mutations? The BCR-ABL1 T315I paradigm. Cancer.

[b38] Gillies RJ, Verduzco D, Gatenby RA (2012). Evolutionary dynamics of carcinogenesis and why targeted therapy does not work. Nature Reviews Cancer.

[b39] Gorbunova V, Seluanov A (2009). Coevolution of telomerase activity and body mass in mammals: from mice to beavers. Mechanisms of Ageing and Development.

[b40] Greaves M, Maley CC (2012). Clonal evolution in cancer. Nature.

[b41] Green DR, Ferguson T, Zitvogel L, Kroemer G (2009). Immunogenic and tolerogenic cell death. Nature Review Immunology.

[b42] Hanahan D, Weinberg RA (2011). Hallmarks of cancer: the next generation. Cell.

[b43] zur Hausen HZ (2009). The search for infectious causes of human cancers: where and why (Nobel lecture). Angewandte Chemie International Edition.

[b44] Huang Q, Li F, Liu X, Li W, Shi W, Liu FF, O'Sullivan B (2011). Caspase 3-mediated stimulation of tumor cell repopulation during cancer radiotherapy. Nature Medicine.

[b107] International network of cancer genome projects Nature.

[b45] Iwami S, Haeno H, Michor F (2012). A race between tumor immunoescape and genome maintenance selects for optimum levels of (epi)genetic instability. PLoS Computational Biology.

[b46] Iwasa Y, Nowak MA, Michor F (2006). Evolution of resistance during clonal expansion. Genetics.

[b47] Komarova NL, Wodarz D (2005). Drug resistance in cancer: principles of emergence and prevention. Proceedings of the National Academy of Sciences of the United States of America.

[b48] Leary RJ, Lin JC, Cummins J, Boca S, Wood LD, Parsons DW, Jones S (2008). Integrated analysis of homozygous deletions, focal amplifications, and sequence alterations in breast and colorectal cancers. Proceedings of the National Academy of Sciences of the United States of America.

[b49] Lee HO, Silva AS, Concilio S, Li YS, Slifker M, Gatenby RA, Cheng JD (2011). Evolution of tumor invasiveness: the adaptive tumor microenvironment landscape model. Cancer Research.

[b50] van Leeuwen IMM, Edwards CM, Ilyas M, Byrne HM (2007). Towards a multiscale model of colorectal cancer. World Journal of Gastroenterology: WJG.

[b51] Lorz A, Lorenzi T, Hochberg ME, Clairambault J, Perthame B (2012). Populational adaptive evolution, chemotherapeutic resistance and multiple anti-cancer therapies. Mathematical Modelling and Numerical Analysis.

[b52] Ma QC, Ennis CA, Aparicio S (2012). Opening Pandora's Box – the new biology of driver mutations and clonal evolution in cancer as revealed by next generation sequencing. Current Opinion in Genetics and Development.

[b53] Maley CC, Galipeau PC, Finley JC, Wongsurawat VJ, Li X, Sanchez CA, Paulson TG (2006). Genetic clonal diversity predicts progression to esophageal adenocarcinoma. Nature Genetics.

[b54] Mani SA, Guo W, Liao MJ, Eaton EN, Ayyanan A, Zhou AY, Brooks M (2008). The epithelial-mesenchymal transition generates cells with properties of stem cells. Cell.

[b55] Martinez-Quintanilla J, Cascallo M, Fillat C, Alemany R (2009). Antitumor therapy based on cellular competition. Human Gene Therapy.

[b56] Marusyk A, Polyak K (2010). Tumor heterogeneity: causes and consequences. Biochimica et Biophysica Acta (BBA). Reviews on Cancer.

[b57] Marusyk A, Almendro V, Polyak K (2012). Intra-tumour heterogeneity: a looking glass for cancer?. Nature Reviews Cancer.

[b58] Marzac C, Garrido E, Tang R, Fava F, Hirsch P, De Benedictis C, Lapusan S (2011). ABC transporters associated with chemoresistance: transcriptional profiling in extreme cohorts, and their prognostic impact in a cohort of 281 AML patients. Haematologica.

[b59] Merlo LMF, Maley CC (2010). The role of genetic diversity in cancer. Journal of Clinical Investigation.

[b60] Merlo LM, Pepper JW, Reid BJ, Maley CC (2006). Cancer as an evolutionary and ecological process. Nature Reviews Cancer.

[b61] Michor F (2008). Mathematical models of cancer stem cells. Journal of Clinical Oncology.

[b62] Michor F, Hughes TP, Iwasa Y, Branford S, Shah NP, Sawyers CL, Nowak MA (2005). Dynamics of chronic myeloid leukemia. Nature.

[b63] Michor F, Nowak MA, Iwasa Y (2006). Stochastic dynamics of metastasis formation. Journal of Theoretical Biology.

[b64] Miller BE, Miller FR, Wilburn D, Heppner GH (1988). Dominance of a tumour subpopulation line in mixed heterogeneous mouse mammary tumours. Cancer Research.

[b65] Morel AP, Lièvre M, Thomas C, Hinkal G, Ansieau S, Puisieux A (2008). Generation of breast cancer stem cells through epithelial-mesenchymal transition. PLoS ONE.

[b66] Navin N, Krasnitz A, Rodgers L, Cook K, Meth J, Kendall J, Riggs M (2010). Inferring tumor progression from genomic heterogeneity. Genome Research.

[b67] Navin N, Kendall J, Troge J, Andrews P, Rodgers L, McIndoo J, Cook K (2011). Tumour evolution inferred by single-cell sequencing. Nature.

[b68] Nowell PC (1976). The clonal evolution of tumour cell populations. Science.

[b69] Oertel M, Menthena A, Dabeva MD, Shafritz DA (2006). Cell competition leads to a high level of normal liver reconstitution by transplanted fetal liver stem/progenitor cells. Gastroenterology.

[b70] Osborne JM, Walter A, Kershaw SK, Mirams GR, Fletcher AG, Pathmanathan P, Gavaghan D (2010). A hybrid approach to multi-scale modelling of cancer. Philosophical Transactions of the Royal Society A Mathematical, Physical and Engineering Sciences.

[b71] Parmigiani G, Boca S, Lin J, Kinzler KW, Velculescu V, Vogelstein B (2009). Design and analysis issues in genome-wide mutation analysis studies of cancer. Genomics.

[b72] Peto R, Roe FJ, Lee PN, Levy L, Clack J (1975). Cancer and ageing in mice and men. British Journal of Cancer.

[b73] Pienta KJ, McGregor NMC, Axelrod R, Axelrod DE (2008). Ecological therapy for cancer: defining tumors using an ecosystem paradigm suggests new opportunities for novel cancer treatments. Translational Oncology.

[b74] Polyak K, Haviv I, Campbell IG (2009). Co-evolution of tumor cells and their microenvironment. Trends in Genetics.

[b75] Riegman PH, van Veen EB (2011). Biobanking residual tissues. Human Genetics.

[b76] Riester M, Stephan-Otto Attolini C, Downey RJ, Singer S, Michor F (2010). A differentiation-based phylogeny of cancer subtypes. PLoS Computational Biology.

[b77] Roche B, Hochberg ME, Caulin AF, Maley CC, Gatenby RA, Missé D, Thomas F Natural resistance to cancers: a Darwinian hypothesis to explain Peto's paradox. BMC Cancer.

[b78] Romanov GA, Vanyushin BF (1981). Methylation of reiterated sequences in mammalian DNAs. Effects of the tissue type, age, malignancy and hormonal induction. Biochimica et Biophysica Acta.

[b79] Seluanov A, Hine C, Bozzella M, Hall A, Sasahara THC, Ribeiro AACM, Catania KC (2008). Distinct tumor suppressor mechanisms evolve in rodent species that differ in size and lifespan. Aging Cell.

[b80] Shipitsin M, Polyak K (2008). The cancer stem cell hypothesis: in search of definitions, markers and relevance. Laboratory Investigation.

[b81] Sjöblom T, Jones S, Wood LD, Parsons DW, Lin J, Barber TD, Mandelker D (2006). The consensus coding sequences of human breast and colorectal cancers. Science.

[b82] Smith KR, Hanson HA, Mineau GP, Buys SS (2012). Effects of *BRCA1* and *BRCA2* mutations on female fertility. Proceedings of the Royal Society of London Series B-Biological Sciences.

[b83] Solé RV, Deisboeck TS (2004). An error catastrophe in cancer?. Journal of Theoretical Biology.

[b84] Sottoriva A, Vermeulen L, Tavaré S (2011). Modeling evolutionary dynamics of epigenetic mutations in hierarchically organized tumors. PLoS Computational Biology.

[b85] Sprouffske K, Pepper JW, Maley CC (2011). Accurate reconstruction of the temporal order of mutations in neoplastic progression. Cancer Prevention Research.

[b86] Stephan-Otto Attolini C, Cheng Y-K, Beroukhim R, Getz G, Abdel-Wahab O, Levine RL, Mellinghoff IK (2010). A mathematical framework to determine the temporal sequence of somatic genetic events in cancer. Proceedings of the National Academy of Sciences of the United States of America.

[b87] Stephens PJ, Greenman CD, Fu B, Yang F, Bignell GR, Mudie LJ, Pleasance ED (2011). Massive genomic rearrangement acquired in a single catastrophic event during cancer development. Cell.

[b88] Subramanian A, Shackney S, Schwartz R (2012). Inference of tumor phylogenies from genomic assays on heterogeneous samples. Journal of Biomedicine and Biotechnology.

[b89] Summers K, Crespi B (2008). The androgen receptor and prostate cancer: a role for sexual selection and sexual conflict?. Medical Hypotheses.

[b90] Summers K, Crespi BJ (2010). Xmrks the spot: life history tradeoffs, sexual selection and the evolutionary ecology of oncogenesis. Molecular Ecology.

[b91] Takahashi K, Yamanaka S (2006). Induction of pluripotent stem cells from mouse embryonic and adult fibroblast cultures by defined factors. Cell.

[b92] Tamori Y, Bialucha CU, Tian A-G, Kajita M, Huang Y-C, Norman M, Harrison N (2010). Involvement of Lgl and Mahjong/VprBP in cell competition. PLoS Biology.

[b93] Tang R, Hirsch P, Fava F, Lapusan S, Marzac C, Teyssandier I, Pardo J (2009). High Id1 expression is associated with poor prognosis in 237 patients with acute myeloid leukemia. Blood.

[b94] Tarafa G, Prat E, Risques RA, Gonzalez S, Camps J, Grau M, Guino E (2003). Common genetic evolutionary pathways in familial adenomatous polyposis tumors. Cancer Research.

[b95] Thomas F, Elguero E, Brodeur J, Le Goff J, Missé D (2011). Herpes simplex virus type 2 and cancer: a medical geography approach. Infection, Genetics and Evolution.

[b96] Thomas F, Lafferty KD, Brodeur J, Elguero E, Gauthier-Clerc M, Missé D (2012a). Incidence of adult brain cancers is higher in countries where the protozoan parasite *Toxoplasma gondii* is common. Biology Letters.

[b97] Thomas F, Elguero E, Brodeur J, Missé D, Raymond M (2012b). Malignancies and high birthweight in human: which cancers could result from antagonistic pleiotropy?. Journal of Evolutionary Medicine.

[b98] Tomasetti C, Levy D (2010). An elementary approach to modeling drug resistance in cancer. Mathematical Biosciences and Engineering.

[b99] Tomlinson I, Bodmer W (1999). Selection, the mutation rate and cancer: ensuring that the tail does not wag the dog. Nature Medicine.

[b100] Vainchenker W, Delhommeau F, Constantinescu SN, Bernard OA (2011). New mutations and pathogenesis of myeloproliferative neoplasms. Blood.

[b101] Valastyan S, Weinberg RA (2011). Tumor metastasis: molecular insights and evolving paradigms. Cell.

[b102] Vineis P, Berwick M (2006). The population dynamics of cancer: a Darwinian perspective. International Journal of Epidemiology.

[b103] Visvader JE, Lindeman GJ (2008). Cancer stem cells in solid tumours: accumulating evidence and unresolved questions. Nature Reviews Cancer.

[b104] Vittecoq M, Elguero E, Lafferty KD, Roche B, Brodeur J, Gauthier-Clerc M, Missé D (2012). Brain cancer mortality rates increase with *Toxoplasma gondii* seroprevalence in France. Infection, Genetics and Evolution.

[b105] Wood LD, Parsons DW, Jones S, Lin J, Sjoblom T, Leary R, Shen D (2007). The genomic landscapes of human breast and colorectal cancers. Science.

[b117] World Health Organization (2008). Cancer fact sheet number 297.

[b106] Zender L, Spector MS, Xue W, Flemming P, Cordon-Cardo C, Silke J, Fan ST (2006). Identification and validation of oncogenes in liver cancer using an integrative oncogenomic approach. Cell.

